# Supplementary material for: Wisp1 is a circulating factor that stimulates proliferation of adult mouse and human beta cells
Source: Nat Commun. 2020 Nov 25;11:5982. doi: 10.1038/s41467-020-19657-1 (PMC7689468; doi:10.1038/s41467-020-19657-1)
Supplement: Supplementary file 1 — Supplementary Information [file 41467_2020_19657_MOESM1_ESM.pdf]

Supplementary information

for

Wisp1 is a circulating factor that stimulates proliferation of adult mouse and human beta cells

Rebeca Fernandez-Ruiz, Ainhoa García, Yaiza Esteban, Joan Mir, Berta Serra Navarro, Fontcuberta-PiSunyer M, Christophe Broca, Mathieu Armanet, Anne Wojtusciszyn, Vardit Kram, Marian F Young, Josep Vidal, Ramon Gomis, Rosa Gasa

This PDF file includes:

Supplementary Methods, page 2

Supplementary Figures 1 to 8, pages 3-11

Supplementary Tables 1 and 2, pages 12-13

SUPPLEMENTARY METHODS

Bone immunohistochemistry

Femurs from p10 or 11wo mice were decalcified and paraffin-embedded. 6µm longitudinal sections were made. The sections were then deparaffinized and rehydrated. Following antigen retrieval (Unitrieve, Innovex), and quenching of endogenous peroxidase activity with dual endogenous enzyme block (Dako), sections were blocked with 10% normal goat serum for 1 h at 37 °C. Wisp1 rabbit antisera (LF-187, 1:500; from Dr. Larry W. Fisher, NIH) was added to sections and incubated overnight at 4 °C. The samples were then incubated with Super PicTure™ Polymer detection kit (Invitrogen) for 10 min at room temperature and detected with NovaRED™ Peroxidase (HRP) Substrate Kit (Vector laboratories). Mayer's hematoxylin was used as counterstain. Slides were scanned using an Aperio ScanScope slide scanner.

Wisp1 secretion

Cells were isolated from calvaria (skull) bone of p11 and 9wo mice (C57Bl6) as described elsewhere [PMID: 28974711]. Briefly, calvarial osteoblastic cells were obtained from 11 day-old mice by 3 successive 1 mg/ml Collagenase-P trypsin (Roche Diagnostics, Sigma Aldrich; respectively) digestions. Cells were grown in αMEM (Gibco) supplemented with 15% lot-selected non-heat inactivated FBS (Gemini Bio Products), 100U/ml each of penicillin streptomycin (Gibco), 2 mM/ml glutamax, 0.011 µM/ml, 2-mercaptoethanol (Gibco), 10^{-8} M/ml dexamethasone. Cells were plated at a density of 50,000 cells/well and cultured in regular media for five days. Concentration of Wisp1 in the culture media was measured using a commercial ELISA (Quantikine ELISA mouse/rat WISP1/CCN4, R&D systems) and total secreted protein was determined by Pierce™ BCA protein Assay kit (Thermo scientific).

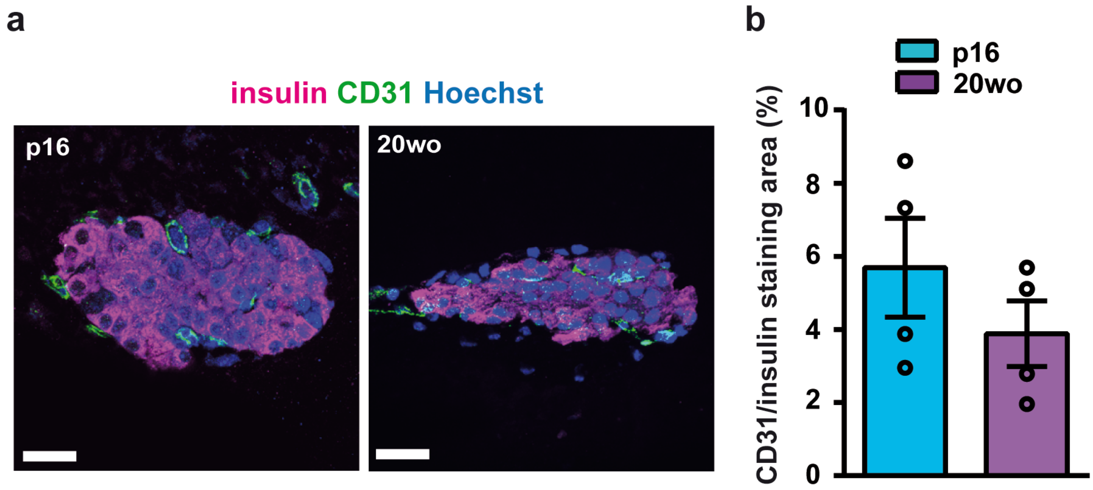

Supplementary Figure 1: Vascularization of 20wo islets transplanted into the anterior chamber of the eye of young and adult C57BL6/J mice. **a** Representative immunofluorescence images showing staining for CD31 (green) and insulin (purple) in 20wo islets transplanted into the anterior chamber of the eye of p16 and 20wo recipients. Nuclei are marked with Hoechst (blue). **b** Quantification of the total area covered by blood vessels in relation to the insulin area in islet grafts transplanted into the anterior chamber of the eye of p16 (blue) and 20wo (purple) recipients. Data are shown as mean ± SEM from n=4 grafts. There is no statistical significance (p=0,3098) between p16 and 20wo recipients using two-tailed Student's t test. Scale bars are 25μm.

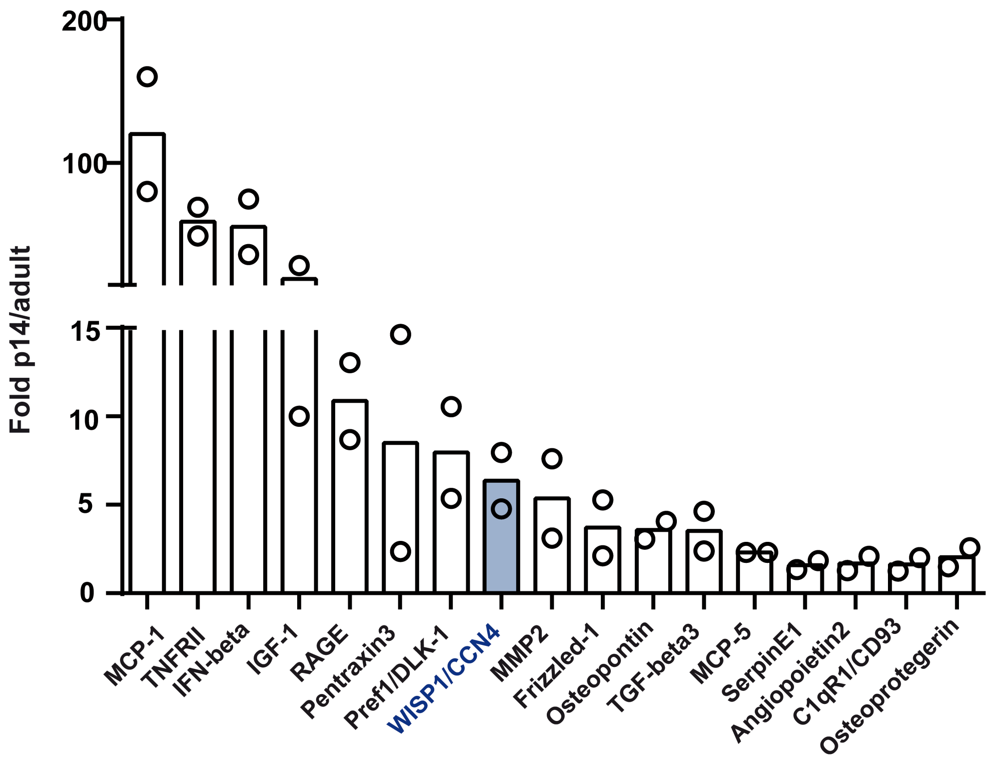

Supplementary Figure 2: Proteins identified using antibody arrays that are more abundant (by at least a 2-fold cut-off) in p14 as compared to 20wo mouse serum. Bars are the mean from n=2 (individual values shown as empty circles).

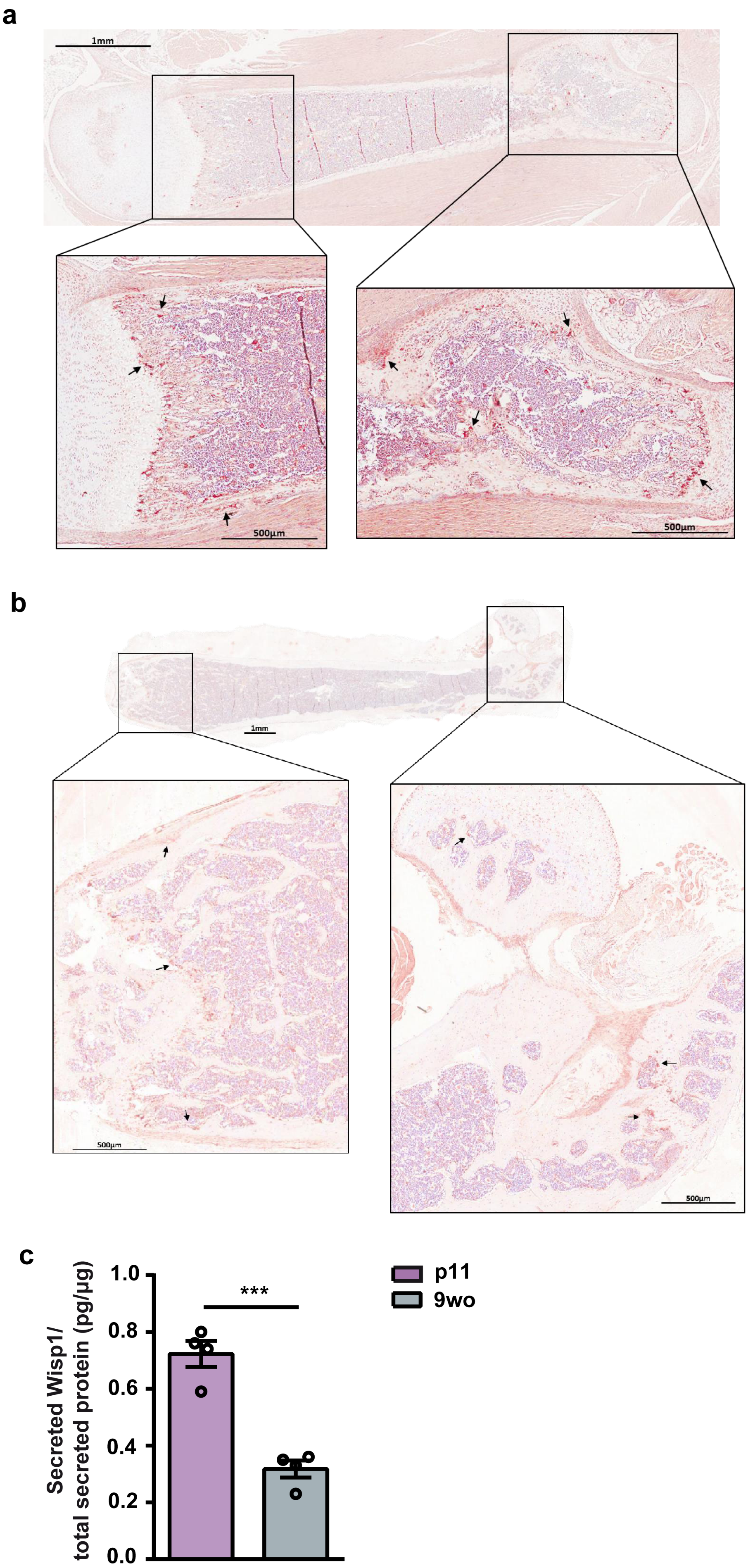
a
1mm
500µm
500µm
b
1mm
500µm
500µm
c
Secreted Wisp1/
total secreted protein (pg/µg)
1.0
0.8
0.6
0.4
0.2
0.0
***
p11
9wo

Supplementary Figure 3: a,b Representative sections of p10 **(a)** and 11wo **(b)** femurs immunohistochemically probed with Wisp1 antisera [LF187; 1:500]. Upper panels low magnification, bar=1 mm; Boxed areas are shown in higher power at lower panels, bar=500µm. Positive staining of Wisp1 is seen in the chondro-osteoblast transition of the primary spongiosa, lining cells surrounding the trabeculi, the periosteal cells as well as by osteocytes already embedded in mineralized matrix. Note that in the p10 bone the staining is more abundant, and the intensity is higher (n=3). **c** Wisp1 production by calvaria cells isolated from p11 and 9wo mice. Wisp1 levels were normalized to total secreted protein. Data are mean ± SE for n=4 independent wells; ***p=0.0003 using two tailed Student's t test.

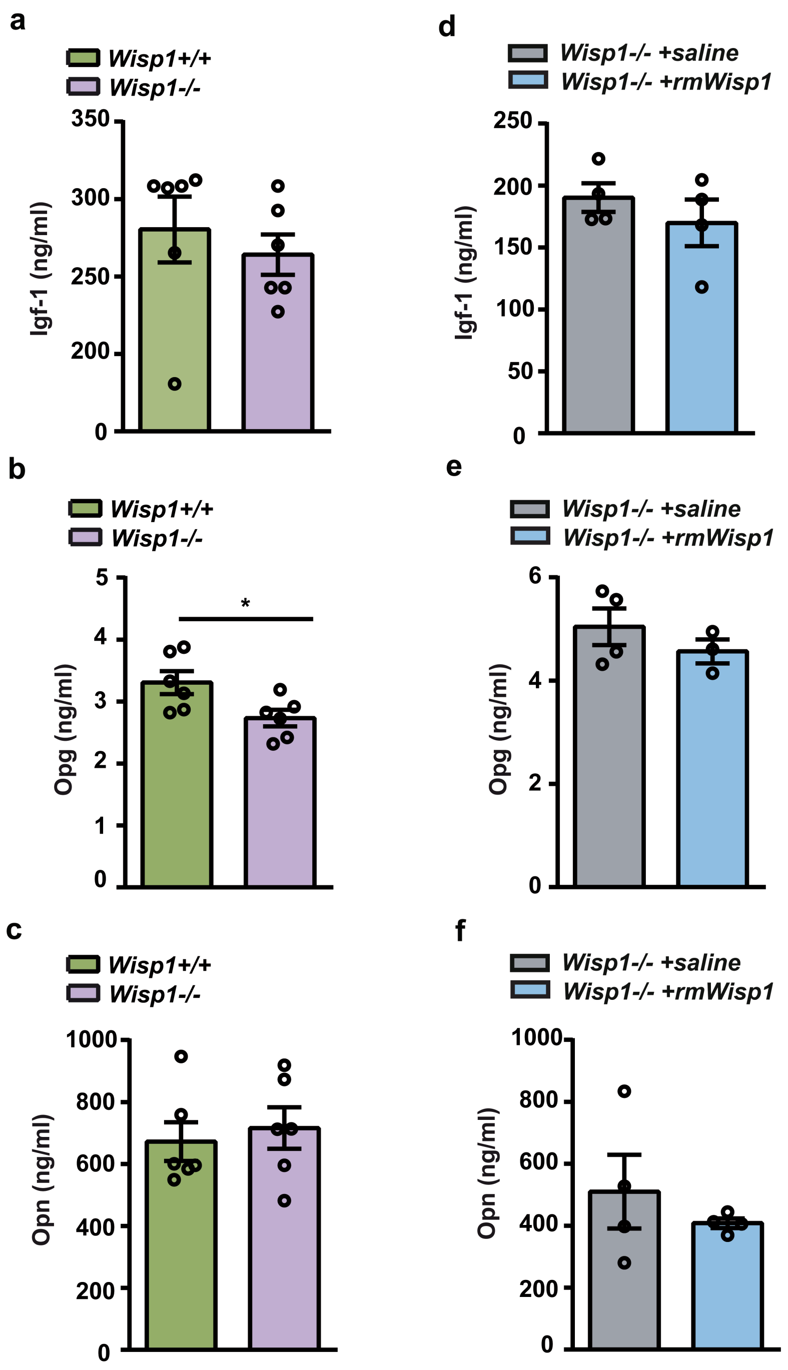

Supplementary Figure 4: a-c Serum levels of Igf-1 (**a**), osteoprogeterin (**b**) and osteopontin (**c**) in p14 Wisp$^{+/+}$ (n=6, green) and Wisp$^{-/-}$ mice (n= 6, purple). **d-f** Serum levels of Igf-1 (**d**), osteoprogeterin (**e**) and osteopontin (**f**) in p12 *Wisp1*$^{-/-}$ mice treated with saline (n=4, grey) or with recombinant mouse Wisp1 (n=4, blue) for three days (from p9 to p11). Data shown are mean ± SEM for the indicated n. *p=0.03 using two-tailed Student's t test. No statistical significance was found for any of the other comparisons.

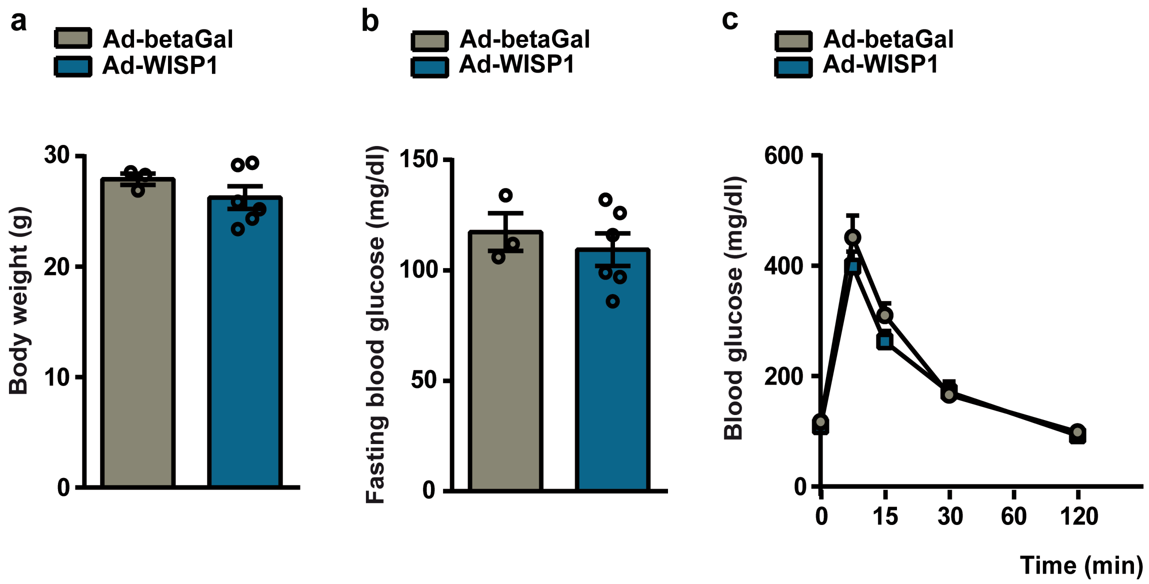

Supplementary Figure 5: Adult C57BL6/J mice were injected via the tail vein with adenoviruses encoding human WISP1 (n=6) or beta-galactosidase (n=3). **a** Body weight fourteen days after adenoviral injection. **b** Fasting blood glucose levels 14 days after adenoviral injection. **c** Intraperitoneal glucose tolerance test 14 days after adenoviral injection. All data are represented as mean ± SEM. No statistical significance was found for any of the studied parameters using two-tailed Student's t-test (a,b) or two-way ANOVA (c).

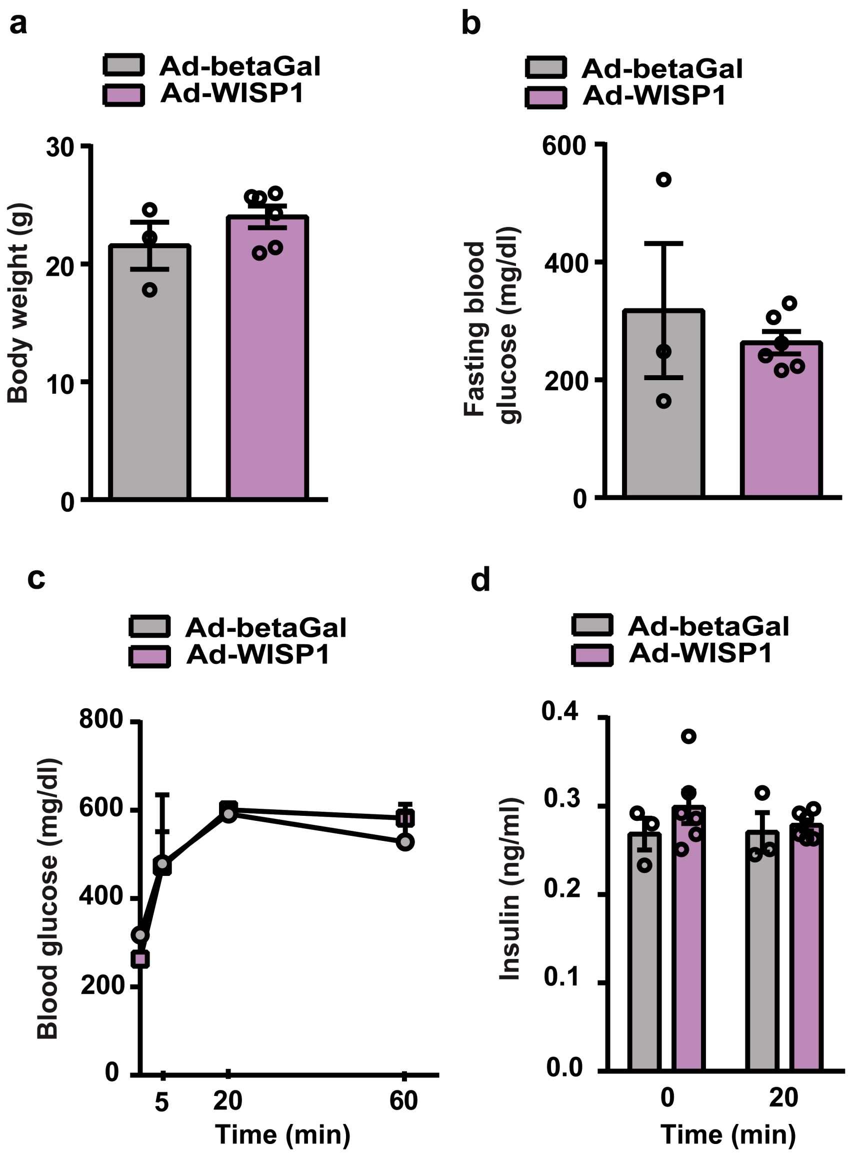

Supplementary Figure 6: Stz-induced diabetic C57BL6/J mice were injected via the tail vein with adenoviruses encoding human WISP1 (n=6) or beta-galactosidase (n=3). **a** Body weight fourteen days after adenoviral injection. **b** Fasting blood glucose levels at day 14 after adenoviral injection. **c** Intraperitoneal glucose tolerance test in fasted diabetic mice fourteen days after adenoviral injection. **d** Plasma insulin levels in fasted diabetic mice at time 0 and 20 min after injection of an intraperitoneal glucose bolus. All data represented are mean ± SEM for the indicated n. No statistical significance was found for any of the studied parameters using two-tailed Student's t-test (a,b,d) or or two-way ANOVA (c).

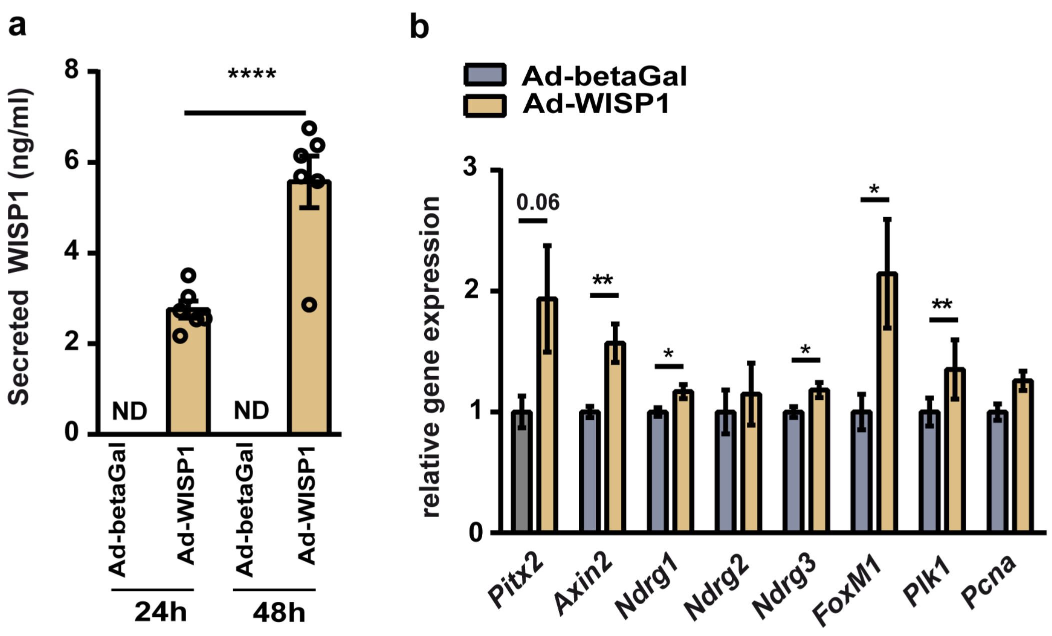

Supplementary Figure 7: NIH3T3 cells were infected with recombinant adenoviruses encoding human WISP1 (Ad-WISP1) or beta-galactosidase (Ad-betaGal) and then co-cultured for 24 or 48h with adult mouse islets. **a** Human WISP1 concentration in the co-culture media was measured by ELISA. Data are shown as mean ± SEM from n=6 independent experiments. Human WISP1 was not detectable (ND) in Ad-betaGal co-cultures. **b** Gene expression measured by qPCR of genes related to WISP1 signaling and cell cycle in mouse islets co-cultured for 48h with NIH3T3 cells previously infected with the indicated adenoviruses. Data are shown as mean ± SEM from n=12 islet batches (except for *Ndrg2*, n=4) corresponding to two independent islet isolation experiments. *p<0.05, **p<0.005, **** p<0.0001 using two-tailed Student's t-test.

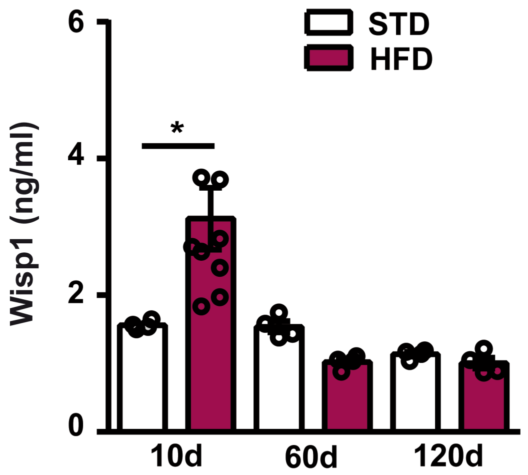

Supplementary Figure 8: 6wo male C57BL6/J mice were fed a 60% high fat diet (HFD, purple bars) or standard chow diet (STD, empty bars) for 120 days. Ad libitum plasma levels of Wisp1 were measured with ELISA at days 10, 60 and 120. Data are shown as mean ± SEM from n=9 mice in the HFD/10d group and n=4 for the rest. *p=0.02 using two-way ANOVA.

Supplementary Table 1. List of oligonucleotides

Gene	Sequence	For
Wisp1Neo	GTG CTT TAC GGT ATC GCC GCT	Genotyping
Wisp1Exon2L	ACC CCC ACA ACA ATG ACC T	Genotyping
Wisp1Exon2R	AGC TGC TGG GCA CAT ATC TT	Genotyping
mTbp-fw	ACCCTTCACCAATGACTCCTATG	qRT-PCR
mTbp-rv	ATGATGACTGCAGCAAATCGC	qRT-PCR
m/hCyr61-CCN1-fw	AAAGGCAGCTCACTGAAG	qRT-PCR
m/hCyr61-CCN1-rv	GCCGGTATTTCTTGACAC	qRT-PCR
m/hCtgf-CCN2-fw	TTCCCGAGAAGGGTCAAGCT	qRT-PCR
m/hCtgf-CCN2-rv	TCCTTGGGCTCGTCACACA	qRT-PCR
mWisp1-CCN4-fw	GGTATCTCCACTCGGATCTCT	qRT-PCR
mWisp1-CCN4-rv	CCCTGCCTTGATGTGTAGTT	qRT-PCR
hWISP1 -fw	AGTGGGTATGTGAGGACGA	qRT-PCR
hWISP1 -rv	GCTTGTGTAGGCTATGCAGTTC	qRT-PCR
mNdrg1-fw	CGATGTTCAGGAGCAGGATATT	qRT-PCR
mNdrg1-rv	CGTGATACGTGAGGATGACAG	qRT-PCR
mNdrg2-fw	CACTCTGTGGAGACACCTTATG	qRT-PCR
mNdrg2-rv	TGGCTGGAAGCAAGACTTATAG	qRT-PCR
mNdrg3-fw	GCTCTTCCTGGGTTCCTATAATG	qRT-PCR
mNdrg3-rv	CACCACCAGTAAGGTAGAACAC	qRT-PCR
mPitx2-fw	ATAAGGGCCAGCAAGGAAAG	qRT-PCR
mPitx2-rv	AGCTGCTGGCTAGTGAAATG	qRT-PCR
mAxin2-fw	TGGAGAGTGAGCGGCAGAGC	qRT-PCR
mAxin2 -rv	TGGAGACGAGCGGGCAGAC	qRT-PCR
mFoxm1-fw	TGAGGGTCAAAGCTTGCGAT	qRT-PCR
mFoxm1-rv	TCTGATGTTTCACTCGGGGC	qRT-PCR
mPolo like kinase 1-fw	CTTCGCCAAATGCTTCGAGAT	qRT-PCR
mPolo like kinase 1-rv	TAGGCTGCGGTGAATTGAGAT	qRT-PCR
mPcna-fw	ATCGTGAATCGGGGG	qRT-PCR
mPcna-rv	AAACATGGTGGCGGA	qRT-PCR

Supplementary Table 2. List of antibodies

WB: western blot; IF:immunofluorescence: IHC: immunohistochemistry

PRIMARY ANTIBODIES	RAISED IN	DILUTION	SOURCE
insulin	guinea pig	1/500 (IF)	DAKO #A0564
ki67	rabbit	1/200 (IF)	Thermo Scientific #MA5-14520
Phosphohistone H3 (S10)	rabbit	1/500 (IF)	Millipore #06-570
p-AKT (S473)	rabbit	1/1000 (WB)	Cell Signaling #9271S
AKT (pan) (C67E7)	rabbit	1/1000 (WB)	Cell Signaling #4691
Actin	rabbit	1/1000 (WB)	SIGMA #A2066
Tubulin	mouse	1/1000 (WB)	SIGMA #T6074
CD31/PECAM-1	rabbit	1/1000 (IF)	Abcam #Ab28364
Wisp1/LF-187	rabbit	1/500 (IHC)	Dr. Larry W. Fisher, NIH

SECONDARY ANTIBODIES	RAISED IN	DILUTION	SOURCE
Alexa Fluor®555 anti-guinea pig	donkey	1/400 (IF)	Invitrogen #A21435
Alexa Fluor®488 anti-rabbit	donkey	1/400 (IF)	Jackson Immunoresearch #711-546-152
Anti-Rabbit IgG Peroxidase	donkey	1/5000 (WB)	GE Healthcare #NA934
Anti-Mouse IgG Peroxidase	sheep	1/5000 (WB)	GE Healthcare #NA931
Normal Donkey serum		5% (IF)	Jackson Immunoresearch #017-000-121
Normal Goat Serum		5% (IF)	Jackson Immunoresearch #005-000-121
