# Supplementary material for: Wisp1 is a circulating factor that stimulates proliferation of adult mouse and human beta cells
Source: Nat Commun. 2020 Nov 25;11:5982. doi: 10.1038/s41467-020-19657-1 (PMC7689468; doi:10.1038/s41467-020-19657-1)
Supplement: Supplementary file 3 — Description of Additional Supplementary Files [file 41467_2020_19657_MOESM3_ESM.docx]

**Description of additional supplementary files**

for

**Wisp1 is a circulating factor that stimulates proliferation of adult mouse and human beta cells**

Rebeca Fernandez-Ruiz, Ainhoa García, Yaiza Esteban, Joan Mir, Berta Serra Navarro, Fontcuberta-PiSunyer M, Christophe Broca, Mathieu Armanet, Anne Wojtusciszyn, Vardit Kram, Marian F Young^,^  Josep Vidal, Ramon Gomis, Rosa Gasa

This document includes Figure legends for Supplementary Videos 1-10

File name: **Supplementary video 1**

Description: Vascularization of 20wo mouse islets transplanted into the anterior chamber of the eye of a p16 mouse. Video shows a reconstruction in AVI format of 7-10 consecutive frames of *in vivo* intravital microscopy, depicting insulin in green (CFDA labeling) and blood vessels in red (dextrane).

File name: **Supplementary video 2**

Description: Vascularization of 20wo mouse islets transplanted into the anterior chamber of the eye of a p16 mouse. Video shows a reconstruction in AVI format of 7-10 consecutive frames of *in vivo* intravital microscopy, depicting insulin in green (CFDA labeling) and blood vessels in red (dextrane).

File name: **Supplementary video 3**

Description: Vascularization of 20wo mouse islets transplanted into the anterior chamber of the eye of a p16 mouse. Video shows a reconstruction in AVI format of 7-10 consecutive frames of *in vivo* intravital microscopy, depicting insulin in green (CFDA labeling) and blood vessels in red (dextrane).

File name: **Supplementary video 4**

Description: Vascularization of 20wo mouse islets transplanted into the anterior chamber of the eye of a 20wo mouse. Video shows a reconstruction in AVI format of 7-10 consecutive frames of *in vivo* intravital microscopy, depicting insulin in green (CFDA labeling) and blood vessels in red (dextrane).

File name: **Supplementary video 5**

Description: Vascularization of 20wo mouse islets transplanted into the anterior chamber of the eye of a 20wo mouse. Video shows a reconstruction in AVI format of 7-10 consecutive frames of *in vivo* intravital microscopy, depicting insulin in green (CFDA labeling) and blood vessels in red (dextrane).

File name: **Supplementary video 6**

Description: Vascularization of 20wo mouse islets transplanted into the anterior chamber of the eye of a 20wo mouse. Video shows a reconstruction in AVI format of 7-10 consecutive frames of *in vivo* intravital microscopy, depicting insulin in green (CFDA labeling) and blood vessels in red (dextrane).

File name: **Supplementary video 7**

Description: Vascularization of 20wo mouse islets transplanted into the anterior chamber of the eye of a p16 *Wisp1*^+^/^+^ recipient. Video shows a reconstruction in AVI format of 7-10 consecutive frames of *in vivo* intravital microscopy, depicting insulin in green (CFDA labeling) and blood vessels in red (dextrane).

File name: **Supplementary video 8**

Description: Vascularization of 20wo mouse islets transplanted into the anterior chamber of the eye of a p16 *Wisp1*^+^/^+^ recipient. Video shows a reconstruction in AVI format of 7-10 consecutive frames of *in vivo* intravital microscopy, depicting insulin in green (CFDA labeling) and blood vessels in red (dextrane).

File name: **Supplementary video 9**

Description: Vascularization of 20wo mouse islets transplanted into the anterior chamber of the eye of a p16 *Wisp1*^-^/^-^ recipient. Video shows a reconstruction in AVI format of 7-10 consecutive frames of *in vivo* intravital microscopy, depicting insulin in green (CFDA labeling) and blood vessels in red (dextrane).

File name: **Supplementary video 10**

Description: Vascularization of 20wo mouse islets transplanted into the anterior chamber of the eye of a p16 *Wisp1*^-^/^-^ recipient. Video shows a reconstruction in AVI format of 7-10 consecutive frames of *in vivo* intravital microscopy, depicting insulin in green (CFDA labeling) and blood vessels in red (dextrane).
