# Supplementary material for: Wisp1 is a circulating factor that stimulates proliferation of adult mouse and human beta cells
Source: Nat Commun. 2020 Nov 25;11:5982. doi: 10.1038/s41467-020-19657-1 (PMC7689468; doi:10.1038/s41467-020-19657-1)
Supplement: Supplementary file 14 — Reporting Summary [file 41467_2020_19657_MOESM14_ESM.pdf]

nature research

Corresponding author(s): Rosa Gasa

Last updated by author(s): Mar 8, 2019

Reporting Summary

Nature Research wishes to improve the reproducibility of the work that we publish. This form provides structure for consistency and transparency in reporting. For further information on Nature Research policies, seeAuthors & Referees and theEditorial Policy Checklist .

Statistics

For all statistical analyses, confirm that the following items are present in the figure legend, table legend, main text, or Methods section.

n/a	Confirmed	
[ ]	[x]	The exact sample size (*n*) for each experimental group/condition, given as a discrete number and unit of measurement
[ ]	[x]	A statement on whether measurements were taken from distinct samples or whether the same sample was measured repeatedly
[ ]	[x]	The statistical test(s) used AND whether they are one- or two-sided<br>*Only common tests should be described solely by name; describe more complex techniques in the Methods section.*
[x]	[ ]	A description of all covariates tested
[x]	[ ]	A description of any assumptions or corrections, such as tests of normality and adjustment for multiple comparisons
[ ]	[x]	A full description of the statistical parameters including central tendency (e.g. means) or other basic estimates (e.g. regression coefficient) AND variation (e.g. standard deviation) or associated estimates of uncertainty (e.g. confidence intervals)
[ ]	[x]	For null hypothesis testing, the test statistic (e.g. *F*, *t*, *r*) with confidence intervals, effect sizes, degrees of freedom and *P* value noted<br>*Give P values as exact values whenever suitable.*
[x]	[ ]	For Bayesian analysis, information on the choice of priors and Markov chain Monte Carlo settings
[x]	[ ]	For hierarchical and complex designs, identification of the appropriate level for tests and full reporting of outcomes
[x]	[ ]	Estimates of effect sizes (e.g. Cohen's *d*, Pearson's *r*), indicating how they were calculated

Our web collection on statistics for biologists contains articles on many of the points above.

Software and code

Policy information about availability of computer code

Data collection	Confocal image acquisition was performed with Leica LAS AF v2.7.3.9723.
Data analysis	Data were analysed with GraphPad Prism (Prism version 6.00 for Windows, GraphPad software, www.graphpad.com) and Microsoft Office Excel 2007.<br>Image J v1.50d Software (Wayne Rayband, National Institutes of Health (NIH)) was used for western blot and immunoimaging analysis. All information about software is reported in the materials and methods section of the manuscript.

For manuscripts utilizing custom algorithms or software that are central to the research but not yet described in published literature, software must be made available to editors/reviewers. We strongly encourage code deposition in a community repository (e.g. GitHub). See the Nature Research guidelines for submitting code & software for further information.

Data

Policy information about availability of data

All manuscripts must include a data availability statement. This statement should provide the following information, where applicable:

- Accession codes, unique identifiers, or web links for publicly available datasets
- A list of figures that have associated raw data
- A description of any restrictions on data availability

Provide your data availability statement here.

Field-specific reporting

Please select the one below that is the best fit for your research. If you are not sure, read the appropriate sections before making your selection.

Life sciences study design

All studies must disclose on these points even when the disclosure is negative.

Sample size	Sample sizes were not predetermined, but chosen to ensure adequate power based on the available literature and protocols in the field. Sample sizes differed based on the type of experiment and source material. For in vivo mouse experiments, sample size ranged from 3-13 individual mice. For whole islet studies, sample size ranged from 15-100 individual islets. The number of human islet and blood samples were limited by the availability of human donors. We used 8 independent human islet batches. We measured human plasma WISP1 levels in 11 children and 14 adult subjects.
Data exclusions	Regarding the STZ-induced diabetic mouse model, two mice were excluded due to the fact that they did not reach a hyperglycemic state, defined as glycemia equal or higher than 350mg/dl.
Replication	All the data presented in this work were reproduced, and the exact number of replicates for each experiment is indicated in its correspondant figure legend.
Randomization	For all in vivo experiments, mice were chosen randomly from the age-/sex-matched colonies. All in vitro experiments with mouse islets were performed with randomly selected islets. Human islets from each batch were randomly divided into the different experimental conditions of the experiment.
Blinding	Investigators were not blinded when obtaining the data. During immunofluroescence analysis, investigators were blinded.

Reporting for specific materials, systems and methods

We require information from authors about some types of materials, experimental systems and methods used in many studies. Here, indicate whether each material, system or method listed is relevant to your study. If you are not sure if a list item applies to your research, read the appropriate section before selecting a response.

Materials & experimental systems

n/a	Involved in the study	
☐	☒	Antibodies
☐	☒	Eukaryotic cell lines
☒	☐	Palaeontology
☐	☒	Animals and other organisms
☐	☒	Human research participants
☒	☐	Clinical data

Methods

n/a	Involved in the study	
☒	☐	ChIP-seq
☒	☐	Flow cytometry
☒	☐	MRI-based neuroimaging

Antibodies

Antibodies used	Primary antibodies:<br>Guinea Pig anti-insulin (DAKO #A0564)<br>Rabbit anti-ki67 (SP6) (Thermo Scientific #MA5-14520)<br>Rabbit anti-phosphohistone H3 (S10) (Millipore #06-570)<br>Rabbit anti-phospho Akt (Ser473) (Cell Signaling #9271S)<br>Rabbit anti-Akt (pan-C67E7) (Cell Signaling #4691)<br>Rabbit anti-actin (SIGMA #A2066)<br>Rabbit anti-tubulin (SIGMA #T6074)<br>Rabbit anti-CD31/PECAM (Abcam #Ab28364)<br>Rabbit anti-Wisp1/LF-187 (Dr Larry W. Sisher, National Institutes of Health)<br>Secondary antibodies:<br>Alexa Fluor®555 anti-guinea pig (Invitrogen #A21435)<br>Alexa Fluor®468 anti-rabbit (Jackson Immunoresearch #711-546-152)<br>Anti-Rabbit IgG Peroxidase (GE Healthcare #NA934)<br>Anti-Mouse IgG Peroxidase (GE Healthcare #NA931)<br>Normal Donkey serum (Jackson Immunoresearch #017-000-121)<br>Normal Goat serum (Jackson Immunoresearch #005-000-121)<br><br>The same information and dilutions used for each antibody are provided in Supplementary Table 2.
Validation	-Validation of Rabbit anti-Wisp1/LF-187 was performed at Dr Larry W. Sisher´s lab, in the National Institutes of Health, Bethesda.<br>-Validation of Guinea Pig anti-insulin is provided in Pardo FN et al, Diabetologia 2012 (DOI 10.1007/s00125-012-2696-9).<br>-The rabbit anti-ki67 antibody is validated for IF in the supplier´s website: https://www.thermofisher.com/antibody/product/

Ki-67-Antibody-clone-SP6-Recombinant-Monoclonal/MA5-14520.
-The rabbit anti-phosphohistone H3 is validated for ICC, WB and IP in the supplier´s website: https://www.merckmillipore.com/ES/es/product/Anti-phospho-Histone-H3-Ser10-Antibody-Mitosis-Marker,MM_NF-06-570?ReferrerURL=https%3A%2F%2Fwww.google.com%2F#.
-Rabbit anti-phospho Akt (Ser473) has been cited 4432 times and is validated for WB, IP, IF and F as listed in the website: https://www.cellsignal.com/products/primary-antibodies/phospho-akt-ser473-antibody/9271?Ntk=Products&Ntt=9271.
-Rabbit anti-Akt (pan-C67E7) has been cited 2115 times and is validated for WB, IP, IHC, IF and F as listed in the website:https://www.cellsignal.com/products/primary-antibodies/akt-pan-c67e7-rabbit-mab/4691?Ntk=Products&Ntt=4691.
-Rabbit anti-actin has been cited 2563 times and is validated for WB, IHC and IF as listed in the website:https://www.sigmaaldrich.com/catalog/search?term=A2066&interface=All&N=0&mode=partialmax&lang=es&region=ES&focus=product.
-Rabbit anti-tubulin has been cited 962 times and is validated for WB, IHC and IP as listed in the website: https://www.sigmaaldrich.com/catalog/product/sigma/t6074?lang=es&region=ES.
-Rabbit anti-CD31/PECAM has been cited 1173 times and is validated for IHC-P as listed in the website: https://www.abcam.com/cd31-antibody-ab28364.html.

Eukaryotic cell lines

Policy information about cell lines

Cell line source(s): NIH-3T3 were stored in our laboratory for many years, and the source is unknown.

Authentication: None of the cell lines used were authenticated.

Mycoplasma contamination: Negative for mycoplasm.

Commonly misidentified lines (See ICLAC register): No commonly misidentified cell lines were used in the study.

Animals and other organisms

Policy information about studies involving animals; ARRIVE guidelines recommended for reporting animal research

Laboratory animals: All animal procedures were approved by the Animal Ethics/Research Committee of the University of Barcelona. Principles of laboratory animal care were followed (European and local government guidelines). Animals were maintained on standard light/dark cycle and had ad libitum access to chow food and water. All mice involved in this sudy were bred at the barrier animal facility of the University of Barcelona.
C57BL6/J and immunocompromised NSG-SCID mice were from Jackson Laboratories; Wisp1 knockout mice (Wisp1-/-) were genotyped as described in the methods section. All adult mice used in the experiments were male. Pre-weaning mice were both male and female (stated in the Methods section of the manuscript).

Wild animals: No wild animals were used.

Field-collected samples: No field-collected samples were used.

Ethics oversight: All animal procedures were approved by the Animal Ethics/Research Committee of the University of Barcelona.

Note that full information on the approval of the study protocol must also be provided in the manuscript.

Human research participants

Policy information about studies involving human research participants

Population characteristics: Human islets were obtained from 8 cadaveric donors (males and females) with an average age of 54.1±3.7 years and BMI of 24.7 ±1.4 kg/m2.
Human plasma was obtained from 11 clinically healthy children, aged 2-5 years, and from 14 clinically healthy adult men, aged 28-45 years.

Recruitment: We did not recruit human participants. All human islet samples are isolated from cadaveric donors at islet isolation center in CHU, Montpelier. Clinically healthy children and adults were recruited at the Hospital Infantil Sant Joan de Deu and the Hospital Clinic-IDIBAPS Biobank (both located in Barcelona). None selection bias is present.

Ethics oversight: Experiments involving human islets were performed in agreement with the local ethic committee (CHU, Montpellier) and the institutional ethical committee of the French Agence de la Biomédecine (DC Nos. 2014-2473 and 2016-2716). Informed consent was obtained for all donors.
Human blood samples were obtained from the biobanks of the Hospital Infantil Sant Joan de Deu and Hospital Clinic-IDIBAPS. The ethical committees from both hospitals approved the study protocol and sample cession. Informed consent was obtained from all blood donors or from their legal representative.

Note that full information on the approval of the study protocol must also be provided in the manuscript.
